# Downregulation of Salivary Proteins, Protective against Dental Caries, in Type 1 Diabetes

**DOI:** 10.3390/proteomes9030033

**Published:** 2021-07-19

**Authors:** Eftychia Pappa, Konstantinos Vougas, Jerome Zoidakis, William Papaioannou, Christos Rahiotis, Heleni Vastardis

**Affiliations:** 1 Department of Operative Dentistry, School of Dentistry, National and Kapodistrian University of Athens, 11527 Athens, Greece; craxioti@dent.uoa.gr; 2 Proteomics Laboratory, Biomedical Research Foundation Academy of Athens, 11527 Athens, Greece; kvougas@bioacademy.gr (K.V.); izoidakis@bioacademy.gr (J.Z.); 3 Department of Preventive and Community Dentistry, School of Dentistry, National and Kapodistrian University of Athens, 11527 Athens, Greece; vpapaio@dent.uoa.gr; 4 Department of Orthodontics, School of Dentistry, National and Kapodistrian University of Athens, 11527 Athens, Greece; h.vastardis@gmail.com

**Keywords:** saliva, proteome, caries, diabetes, bioinformatics, proteomics

## Abstract

Saliva, an essential oral secretion involved in protecting the oral cavity’s hard and soft tissues, is readily available and straightforward to collect. Recent studies have analyzed the salivary proteome in children and adolescents with extensive carious lesions to identify diagnostic and prognostic biomarkers. The current study aimed to investigate saliva’s diagnostic ability through proteomics to detect the potential differential expression of proteins specific for the occurrence of carious lesions. For this study, we performed bioinformatics and functional analysis of proteomic datasets, previously examined by our group, from samples of adolescents with regulated and unregulated type 1 diabetes, as they compare with healthy controls. Among the differentially expressed proteins relevant to caries pathology, alpha-amylase 2B, beta-defensin 4A, BPI fold containing family B member 2, protein S100-A7, mucin 5B, statherin, salivary proline-rich protein 2, and interleukin 36 gamma were significantly downregulated in poorly-controlled patients compared to healthy subjects. In addition, significant biological pathways (defense response to the bacterium, beta-defensin activity, proline-rich protein activity, oxygen binding, calcium binding, and glycosylation) were deregulated in this comparison, highlighting specific molecular characteristics in the cariogenic process. This analysis contributes to a better understanding of the mechanisms involved in caries vulnerability in adolescents with unregulated diabetes.

## 1. Introduction

Dental caries is a complex, dynamic, and multifactorial disease. Numerous risk factors contribute to dental caries, host susceptibility, dysbiotic microbiota, and frequent intake of dietary sugars [1]. Saliva has been considered an essential biological modulator involved in maintaining oral homeostasis [1]. This biofluid plays a significant role in preventing a regime shift to caries via various mechanisms [2], such as mouth cleaning, aggregation, elimination of microorganisms, buffering capacity, remineralization of tooth tissues, and antimicrobial defense [3].

According to a recent survey by the Global Oral Health Data Bank, tooth caries’ prevalence varies from 49% to 83%. Irrespective of age, dental caries negatively impact almost all individuals. Data gathered from various surveys have shown that adolescents aged 12 to 19 have the highest number of active dental caries, followed by children and adults [4].

Several studies have evaluated whether type 1 diabetes (T1D) increases caries susceptibility [5,6,7,8,9,10,11,12,13,14,15,16]. However, the results of studies that describe the association between diabetes and the incidence and intensity of caries are conflicting [17,18,19]. It has been reported that most patients with T1D have salivary dysfunction as well as alterations in biochemical and microbiological salivary composition when compared to healthy subjects [5,14,20,21]. Proper metabolic regulation of diabetes is considered fundamental for the mitigation of complications [13]. Moreover, youngsters with T1D are shown to have a lower oral hygiene level and are potentially at a higher risk of future oral disease, mainly when their metabolic disorder is uncontrolled [22]. This is confirmed by a recent meta-analysis, showing a high global prevalence of dental caries among children and adolescents with T1D [23]. Further research on the risk of dental caries is necessary to maximize preventive measures and ensure optimal oral health for such vulnerable patients [18,19,24].

Modern proteomic techniques, including mass spectrometry, have shown that saliva analysis can detect the presence or absence of multiple biomarkers, which can act as potential indicators for the early detection, progression monitoring, or response to treatment of many oral and systemic diseases. Furthermore, saliva sample collection is a non-invasive, painless, simple, quick, easy, safe, and inexpensive option compared to collecting other body constituents [23]. Particularly in specific groups, such as children, saliva can be considered an ideal diagnostic fluid since its collection causes minimal patients’ discomfort and guarantees cooperation [25].

The early detection of at-risk youngsters could reduce the incidence of caries lesions per patient and the average cost for prevention and treatment. The number of studies trying to correlate salivary proteins and dental caries has considerably increased [7,24,26,27,28,29,30]. It has been reported that changes in salivary protein compositions are involved in dental caries etiology [26,29,31,32]. These findings suggest the potential use of salivary proteins as biological indicators of dental caries. However, to the best of our knowledge, salivary proteome in relation to caries incidence has not been previously examined in type 1 diabetes.

The aim of this study was to analyze the salivary proteome of patients with type 1 diabetes and detect differentially expressed proteins specific for the occurrence of carious lesions in this population. Thus, it could be possible to identify biological processes and biomarkers characterizing dental caries’ onset and development, and elucidate saliva’s potential caries’ diagnostic ability.

## 2. Materials and Methods 

### 2.1. Ethics Statement

The Medical Ethics Committee approved the study protocol and written consent forms of the Faculty of Medicine of the National and Kapodistrian University of Athens, following the Declaration of Helsinki (ethical approval code: 353/11-01-2017). All experimental methods were performed according to the relevant guidelines and regulations. The study protocol was explained to both parents and children, and informed written consent to participate in the study was obtained from a parent.

### 2.2. Study Design and Clinical Data

Adolescents with diabetes were enrolled in the study from the Diabetic Centre of P&A Kyriakou, Athens, Children’s Hospital, and age-matched controls from the respective pediatric department.

A total of 36 participants were allocated to three groups. Group 1 (G1) consisted of 12 patients with type 1 diabetes with poor glycemic control, group 2 (G2) of 12 patients with satisfactory glycemic control, while the control group (C) comprised 12 healthy participants. Blood glucose concentration was measured with a glycosometer (Accu-Chek Advantage, Roche). The upper limit of fasting glucose that was considered normal was 100 mg/dL. The metabolic control level of diabetes mellitus was determined by the glycosylated haemoglobin, HbA1c, reflecting glycemia levels over the preceding 6–12 week. The percentage of haemoglobin Hb1Ac was determined by the use of the HPLC (HA8140) Instrument. HbA1c values ≥7.5% (58 mmol/mol) indicated poor glycemic control for type 1 diabetes. The control group was established by matching a child without diabetes, who did not have any systemic disease or take medication, to a child with diabetes. The matching criteria were age, gender, city of residence, fluoride exposure, social background (based on parental education level), and oral hygiene routine [15]. A questionnaire analysis determined caries risk factors for all the study participants to eliminate differences between dietary habits, oral hygiene, dental visits, fluoride intake, and social background [13].

All participants were examined by a group of internal medicine physicians during their regular follow-up. During the examination, the endocrinologist, neurologist, and ophthalmologist performed a clinical assessment of complications. Screening for retinopathy, microalbuminuria, and neuropathy took place during the examination. The presence/diagnosis of any diabetic complication was considered an exclusion criterium for this study’s participants. The presence of systemic or oral diseases affecting the salivary glands (such as recurrent aphthous ulcers, Sjogren syndrome, etc.) was an additional exclusion criterium. All participants in the study were requested to report current or previous medication use. The use of any drugs known to induce hyposalivation during the preceding semester was determined as an exclusion criterium. Xerogenic drugs comprised anticholinergics, amphetamines, antidepressants, antihistamines, diuretics, and antihypertensive agents. For the pediatric population studied, the antihistamines were the most common reason for exclusion due to medication use [33].

The participants were examined for dental caries and overall oral health status. According to the WHO caries diagnostic criteria for epidemiological studies, the clinical dental health status was measured using the decayed, missing, and filled teeth (DMFT) index for permanent teeth [34]. Caries examinations were performed under standardized conditions: with an examination light, mouth mirrors, and dental explorers. The caries examination was performed according to the International Caries Detection and Assessment System (ICDAS) and the threshold for active disease set at the D3 level (dentine caries lesion, ICDAS codes ≥4 was used). According to the criteria manual recommendations of the ICDAS committee, the examiner was trained with educational software (ICDAS training software) to use these criteria before the clinical examination of the teeth. Further training was accomplished with a second examiner trained and validated with the ICDAS criteria. Decayed, filled, and missing teeth were recorded (DMFT-WHO caries index).

The plaque and gingival indices (Silness and Loe, 1964) were additionally evaluated. Plaque index (PI) ascertains the thickness of plaque along the gingival margin. PI ≤ 1 was a prerequisite for participation in the study [35]. The gingival index (GI) was simultaneously recorded, and participants with gingival inflammation were excluded from the study. A score below 1 (GI ≤ 1, no bleeding on probing) was a prerequisite for the participants of all three groups [35]. Routine clinical laboratory methods also measured body mass index (BMI), blood pressure, cholesterol values, and details on all clinical parameters shown in Table 1.

### 2.3. Standardized Sample Collection

The composition of saliva varies considerably depending on different conditions [36]. To effectively control potential sources of variability, the following protocol was applied. On the day of the examination, participants were advised not to eat or drink one hour before their scheduled appointment. All saliva samples were collected between 10:00 a.m. and 12:00 p.m. to minimize any inter-individual variation of saliva composition associated with circadian rhythms. Unstimulated whole saliva was collected from the oral cavity’s anterior floor via passive drooling for all participants. In case the participant became stressed or began to cry, the sample was discarded. Collection tubes were stored on ice at all times during the examination. As soon as the sample was collected, it was centrifuged for removal of all cellular debris. The saliva was supplemented with enzyme inhibitors to suppress enzyme activity and protein degradation by adding a total protease inhibitor cocktail (3.6% *v*/*v* protease inhibitors, Roche), and subsequently stored at −80 °C. 

### 2.4. LC-MS Analysis amd Pathway Analysis

A total of 36 saliva samples were acetone precipitated and separated into six batches. Each batch contained six samples, two from each group (G1, G2, & C). The batches were processed separately, and the samples of each set were labeled using iTRAQ (multiplexed isobaric tagging technology for relative quantitation), before high-pH reverse-phase peptide fractionation, and liquid chromatography–mass spectrometry (LC–MS) analysis. All LC–MS experiments were performed on the Dionex Ultimate 3000 UHPLC system coupled with the high-resolution nano-ESI Orbitrap-Elitemass spectrometer (Thermo Scientific). The collected HCD tandem mass spectra were submitted to the cited Tandem search engine [37] implemented on the trans proteomic pipeline (TPP) software version 4.6 for peptide and protein identifications. The significantly deregulated proteins from the previous procedure were imported into QIAGEN’s ingenuity R pathway analysis (IPA). They were analyzed for biological context against the IPA knowledge base (IPA R , QIAGEN Redwood City, www.qiagen.com/ingenuity, accessed on 17 July 2021). For IPA analysis, differentially expressed proteins were considered those with log2ratio *p*-value < 0.05. Suggestions for further biological and potential clinical interventions were obtained from the L1000CDS2 database [38]. The proteomic and pathway analysis is extensively presented in our previous study [39].

### 2.5. LC-MRM Validation

Liquid chromatography-multiple reaction monitoring (LC-MRM) was subsequently performed on selected proteins for validation. The human spectral library was searched using the software Skyline and the peptide atlas repository to identify proteotypic peptides for the 24 chosen proteins for verification [40]. The peptides selected for MRM analysis along with the corresponding transitions are listed in Table S1. The transition with the highest intensity was selected for quantification using isotope labeled peptide standards.

### 2.6. Bioinformatics Analysis

The proteomic datasets derived from the saliva samples of adolescents with regulated, unregulated type 1 diabetes and healthy controls were analyzed [39]. The bioinformatic tools DAVID (v 6.8) (The Database for Annotation, Visualization, and Integrated Discovery) and STRING (v 11.0) (Search Tool for the Retrieval of Interacting Genes/Proteins) were used for functional annotation and interactome analysis of the proteomic findings.

### 2.7. Statistical Analysis

The proteins presenting statistically significant differential expression as per the magnitude of change (*p* < 0.05) were used for the bioinformatic meta-analysis [39].

Non-parametric tests for >2 samples were applied to analyze the three groups’ data since the variables were not normally distributed. Data were analyzed by Chi-square and Kruskal–Wallis tests, using SPSS package (IBM Statistics, V22, Chicago, IL, USA) with a statistical significance (*p* < 0.05). For the DMFT index, data were analyzed by the Kruskal–Wallis test.

## 3. Results

### 3.1. Clinical Characteristics of the Study Population and Dental Caries

The blood glycated hemoglobin (HbA1c) level was assessed and presented normal values in controls (below 5.9%) and ranged from 6 to 12% in type 1 diabetic patients. HbA1c values ≥7.5% indicated poor metabolic control for type 1 diabetes, while values <7.5% were considered good control of the disease. BMI, blood pressure, cholesterol values, and details on all clinical parameters are shown in Table 1. The three investigated groups were similar regarding demographic characteristics, dietary habits, fluoride supplement use, plaque index, and gingival index. However, the results indicated significantly higher caries levels in poorly controlled adolescents with T1D. The average caries indexes were DMFT(G1) 3.9, DMFT(G2) 0.9, DMFT(C) 1.1, *p* < 0.05 (Table 1). The caries index was not found to be significantly different between well-controlled patients and healthy controls.

### 3.2. Molecular Pathways and Differential Expression of Proteins Involved in Dental Caries

The proteomic analysis yielded 22028 peptides that were confidently identified (FDR < 1%) and quantified by the iTRAQ reporter ions. These peptides corresponded to 4876 individual confident protein identifications (FDR < 5%). For the comparative analysis among groups (poorly-regulated T1D patients, well-regulated T1D patients & healthy controls), only the proteins present at a percentage equal to or greater than 70% of the samples (9 ≥ 12) in each group were selected. Thus, the total protein number considered for analysis was reduced to 2031 proteins. Functional classification of these proteins revealed that enzymes and cytokines were the main functional groups of the salivary proteome, as expected. The results of the proteomics analysis are thoroughly presented in a previous study of our group [39].

**Figure 1:**
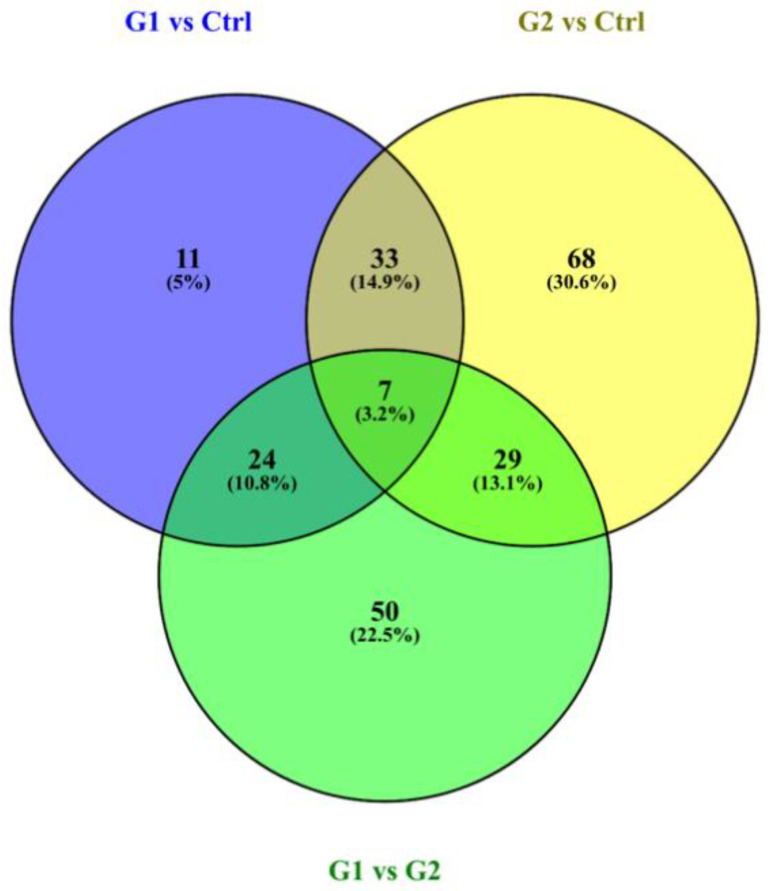
Venn diagram presents the overlap and uniqueness of differentially expressed proteins across the three comparisons. The three circles of the Venn diagram represent the three comparisons. The green circle represents the G1–G2 comparison, the blue represents the G1–Ctrl and the yellow the G2–Ctrl comparison.

The selection of differentially expressed proteins for each pair-wise comparison was performed by applying the log2ratio *p*-value criterium, which corresponded to the magnitude of change for each protein between two groups. All possible comparisons were conducted among the three groups: (G1–C, G2–C, G1–G2). The bioinformatic analysis yielded a total number of 222 differentially expressed proteins across all three comparisons (see Table S2: all the identifications and statistically significant changes, criterion *p*-value of fold change, for all comparisons). In Figure 1, the overlap and uniqueness of differentially expressed proteins are presented across the three comparisons. (see Table S3 for the proteins lists of the Venn Diagram).

The most common categories of the differentially expressed proteins corresponded to secreted proteins (complement system, antibacterial peptides, mucins, and immunoglobulins) and the development of keratinized epithelia (keratins). In Table S4, all the pathways/biological processes across the three comparisons are presented. The following results were obtained from the bioinformatics analysis for differentially expressed pathways involved in dental caries, and the most biologically relevant molecular pathways are shown below (Table 2).

In G1vsC and G1vsG2 comparisons, a more significant number of deregulated molecular pathways associated with caries were identified. From the biological process perspective, the differentially expressed proteins in G1-C comparison were involved in secretion, as expected in saliva proteome. Additionally, they participated in essential mechanisms for caries activity and susceptibilities, such as defense response to the bacterium (gram + and gram), beta-defensin activity, proline-rich protein activity, defense response to fungus oxygen binding, calcium binding, and glycosylation.

**Figure 2:**
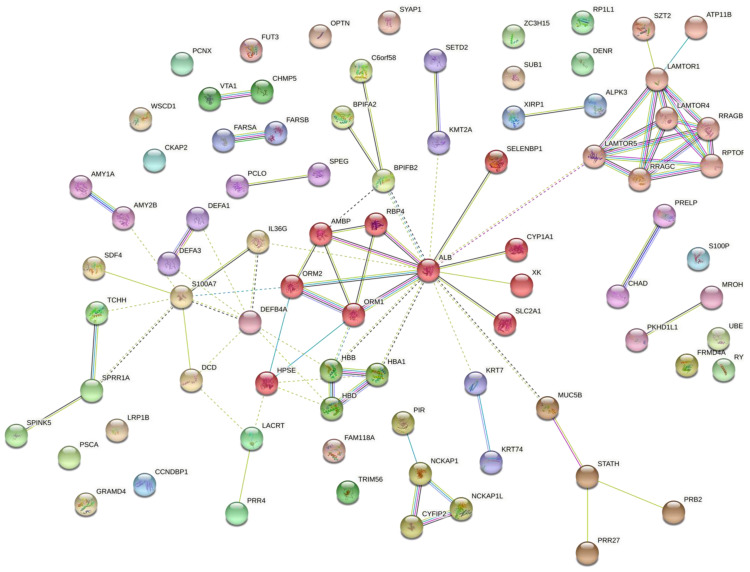
The interaction network for the G1–C comparison indicates that differentially expressed proteins have functional connections. Colored lines between the proteins indicate the various types of interaction evidence. STRING tool was used to draw the protein–protein interactions.

The protein–protein interaction (PPI) network in Figure 2 indicates the functional annotation of the molecular interaction in which differentially expressed proteins participated for the G1–C comparison. S100A7, albumin, defensin 4A, and mucin 5B were critical proteins in these interactions. In Table S5, the list with the annotations in G1–C is presented.

Table 3 presents the expression levels of the most biologically relevant to caries pathology proteins, from the deregulated pathways, across the three comparisons. The following results were obtained from the bioinformatics analysis.

G1–C down: The protein found to be most downregulated was Protein S100-A7 (fold change = −1.69), followed by Alpha-amylase 2B (fold change = −1.55) and Beta defensin 4A (fold change = −1.54). DCD, RBP4, BPIFB2, PRB2, MUC19, MUC5B, IL36G, AMY1A, STATH, ORM2, ALB were also significantly downregulated in this comparison. G1–C up: KRT19, DEFA3, DEFA1, SPRR1A, KRT74, ATP11B, PRR4 were found to be significantly upregulated in this comparison.

G2–C: The majority of the examined proteins were not found to differ significantly in this comparison. PRR4, MUC19, MUC5B, DEF4A and S100A7, DCD were downregulated while APOA2, APOA1, DEFA1, ALB, PRB2 were significantly upregulated.

G1–G2: Following a similar pattern to the G1–C comparison, most examined proteins were downregulated in this pair. Top-down: APOA2 (fold change = −1.44), PRB2 (fold change = −1.62), ALB (fold change = −1.48), ORM2, BPIFB1, C6, C9, SERPINC1, STATH, IL36G, RPB4, S100-A7. Top-up: PRR4, KRT19, KRT74, SPRR1A, ATP11B.

**Figure 3:**
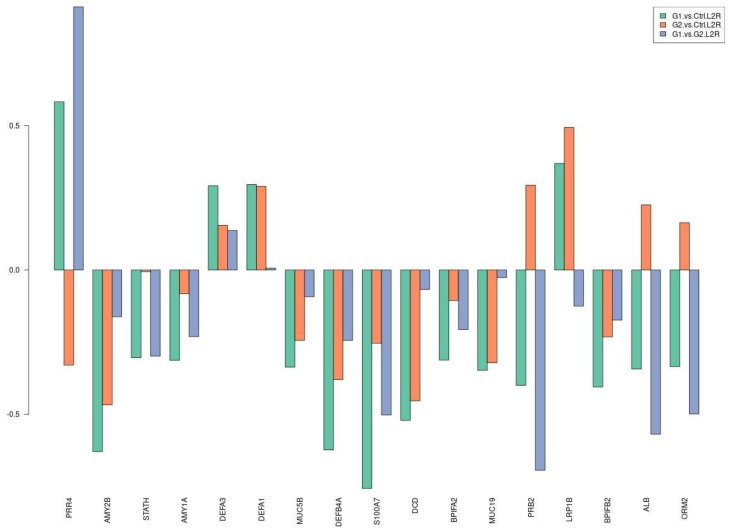
Selection of differentially expressed proteins among the three comparisons. The y axis refers to log2ratio values.

Table 3 and Figure 3 show the **downregulation** of most differentially expressed proteins involved in protective mechanisms for caries activity, both in G1–C and G1–G2 comparisons. Log2ratio values and L2R_pvalues are presented in Table S6.

**Figure 4:**
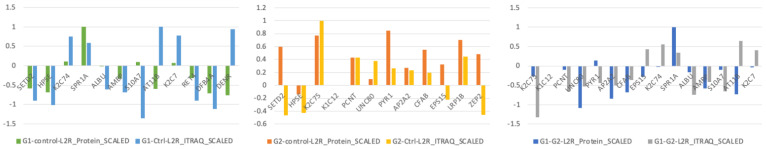
LC-MRM analysis was performed to validate selected proteins across the three comparisons. The *y* axis refers to log2ratio values.

MRM was utilized to validate candidate proteins in salivary proteome. The association between iTRAQ data and MRM validation was established. 12 different samples were pooled to final 100 μg total protein saliva extract for each group. In G1vsG2, 9 out of 12 proteins presented a positive correlation between the itraq and mrm quantitation. Furthermore, 9 out of 12 proteins presented a positive correlation in itraq and mrm quantitation in comparison G1vsCtrl, whereas, in G2vsCtrl, 10 out of 15 presented a positive correlation. Figure 4 and Supplementary Table S7 present iTRAQ versus MRM ratios across the three comparisons.

## 4. Discussion 

### 4.1. Saliva as a Diagnostic Body Fluid 

Saliva surrounds the hard and soft tissues of the oral cavity, comprising organic and inorganic components. Saliva is identified as functionally equivalent to serum, reflecting the body’s physiological state, including hormonal, emotional, nutritional, and metabolic alterations. The collection of saliva is an easy, non-invasive, effortless, chair-side procedure that does not require any special equipment. It ensures patients’ compliance by diminishing the discomfort, which is often associated with blood and urine collection. It is an ideal diagnostic tool for studies conducted on special populations such as children, anxious, handicap, or elderly patients [24]. As the primary host-associated factor, saliva plays an essential role in the dynamic equilibrium between demineralization and remineralization and has been suggested to predict caries’ development [27]. Saliva acts as nature’s primary defense system, and it is vital for protecting the exposed tooth surfaces. It can reverse the exposed tooth surface’s demineralization by simple mechanical rinsing, antimicrobial activity, buffering capacity, calcium phosphate-binding proteins, immune surveillance, and the secretion of antimicrobial peptides [41].

### 4.2. Salivary Alterations in Diabetes

This study analyzed the role of glycemic control of diabetes on caries susceptibility through bioinformatics analysis of salivary proteomes. During adolescence, compliance to health advice is questionable, and adherence to treatment protocols is more difficult to achieve, resulting in frequent metabolic deregulation in young patients. Hyperglycaemia and metabolic deregulation have a significant impact on salivary gland function. Poorly controlled patients secrete significantly less resting and stimulated saliva than well-regulated and healthy controls [13]. These alterations in salivary flow result in higher salivary protein concentration in this group and may be responsible for the increased susceptibility to oral infections and impaired wound healing observed in patients with diabetes. In comparing poorly controlled patients and healthy individuals (G1–C), pathways relevant to caries pathology were significantly deregulated. 

### 4.3. Salivary Proteins and Dental Caries

The salivary proteins, namely the proline-rich proteins (PRP), mucins (MUC), histatins, cystatins, and statherins (STATH), protect the tooth surface, attract calcium ions, and promote remineralization. The most critical factor in preventing dental caries includes remineralizing the initial carious lesion, which requires calcium, phosphate, and fluoride. The amount of calcium and phosphate in the saliva gets supersaturated with calcium and phosphate salts, which have a protective influence on the dental hard tissues [3].

- PRP’s (**PRB2**, **PRR4**)

According to Nobbs et al. (2011) [42], the proline-rich proteins act as a protective mechanism against dental caries. They attach to *Streptococcus mutans* through major adhesion antigen, and this immunological reaction protects the tooth from dental caries. Proline-rich protein decreases the caries incidence by neutralizing the acid production by Streptococci [43]. In our study, PRB2 (proline-rich protein subfamily 2) was significantly downregulated in poorly-controlled patients with T1D (fold change = −1.3 in G1–C, fold change = −1.6 in G1–G2) while PRR4 (proline-rich protein 4) was upregulated in these comparisons (fold change = 1.5 and 1.8 respectively). Intrestingly, levels of PRP-1 and PRP-3 were also found to be increased in caries-susceptible patients [44,45].

- Phosphopeptides (**PRB2**, **STATH**)

Phosphoproteins/phosphopeptides with clusters of acidic residues aid in preventing unwanted precipitation of solid calcium phosphates. The acidic residues, particularly phosphoserine, interact with calcium and stabilize calcium and phosphate clusters, thus significantly contributing to the remineralization process. Results from previous studies indicated strong correlations between high levels of phosphopeptides and the absence of dental caries. In our study, adolescents with uncontrolled T1D presented the most elevated caries experience. In this group, both PRB2 and STATH (fold change = −1.3) were significantly downregulated compared to controlled and healthy participants. According to Cabras et al., statherins, histatins, and PB peptide play a significant role in tooth tissue protection against caries. Therefore, lowering their concentration in the saliva of children who have type 1 diabetes may favor pathological changes in tooth enamel [46]. The above suggest that phosphopeptides might play a significant role in maintaining tooth integrity and protection against cariogenic bacteria [47].

- Albumin (**ALB**)

Salivary albumin acts as a marker for the severity of underlying disease and inflammation. In addition, it has an inhibitory effect on dental caries by preventing enamel demineralization by penetrating the enamel pores [26]. ALB was found to be 1.3 times downregulated in G1–C and 1.5 times down in G1–G2 comparison.

- Mucins (**MUC5B**)

Mucin5B has a protective role against caries, as it has been implicated in cariogenic bacteria’s clearance in the oral cavity by reducing the attachment and biofilm formation of *Streptococcus mutans* [29]. In our analysis, MUC5B was found to be downregulated in adolescents with inadequate metabolic control.

- Histatin-1 and BPI (**BPIFB1**, **BPIFA2**)

BPI fold containing family B member 1 (BPIFB1), which is involved in the antimicrobial humoral response, is found to be downregulated in caries-susceptible young adults and the elderly compared to healthy controls. This result agrees with our study’s findings (BPIFB1/2 fold change = −1.2/−1.3 in G1–C). It highlights the strong correlation between the absence of dental caries and high levels of BPI family member proteins [48].

- Keratins (**KRT74**, **KRT19**)

According to recent studies, a set of keratins is incorporated into mature enamel, and keratin−75 mutations are associated with increased susceptibility to dental caries. [49]. Our analysis showed that KRT74 and KRT19 were significantly upregulated in G1–C comparison, which could be related to increased caries in poorly controlled patients.

- **S100A7** is a calcium- and zinc-binding protein with a prominent role in regulating the immune response and antimicrobial humoral response, and has also been associated with dental caries [31]. Accordingly, it was found to be significantly downregulated (fold change = −1.7) in G1–C comparison.
- Apolipoproteins (**APOA1**, **APOA2**)
  Apolipoproteins have recently been suggested to be particularly relevant to the aging process and longevity by playing crucial human immune functions [50]. Both APOA1 and APOA2 were found to be significantly downregulated in G1–C and G1–G2 comparisons. While their role in periodontitis is recently investigated [51], their possible association with caries susceptibility has yet to be explored.

Decreased saliva secretion in children with unregulated type 1 diabetes causes alterations in salivary protein composition and is significantly related to caries prevalence. Our results confirm that human salivary secretion changes impact caries processes and highlights saliva’s protective function. Poor metabolic control resulted in the deregulation of salivary proteins with a critical biological role in the cariogenic mechanism. The downregulation of most differentially expressed proteins involved in protective mechanisms for caries activity may explain the increased caries incidence in the studied population. Defense response to the bacterium, beta-defensin activity, proline-rich protein activity, oxygen binding, calcium binding, and glycosylation were deregulated in these patients, impairing the process of tooth remineralization and increasing caries risk.

Due to the multifactorial origin of caries, several factors determine a patient’s caries risk. Regarding contributing and confounding effects, the present study considered various parameters to eliminate possible interactions. A questionnaire analysis determined caries risk factors for all the participants in the study. Specific selection criteria excluded potential confounding factors, such as medications, systemic diseases, sugary diet/ frequent snacking, inadequate oral hygiene, and low socioeconomic status for all three groups [1]. The DMFT index describes the burden of dental caries in an individual. WHO adopts it for conducting surveys in oral health assessment for various reasons: its use is simple, valid, reliable, and facilitates comparisons of caries status of the population groups worldwide. During the last decades, the DMFT index has faced intense criticism mainly because its D (decayed) variable, per definition, records only cavitated lesions and ignores incipient lesions that can potentially be reversed in the early stages. Despite these limitations, it remains a reliable tool for caries experience. The present study used it due to its quick and easy way to be registered, especially in community settings [52].

### 4.4. Gingival Inflammation and Diabetes 

PI and GI were assessed as measures of the oral condition of the participants. This emphasizes the relevance of comparing healthy mouths of the different groups of donors, especially for the age groups examined (adolescence). Several studies suggest that diabetes is associated with an increased prevalence, extent, and severity of gingivitis and periodontitis [6,22,53]. Considering the lack of compliance in oral hygiene during adolescence, it was of utmost importance to consider oral plaque and gingival inflammation as an exclusion criterion for all the participants in the study. For that purpose, plaque index and gingival index were recorded by a specialized dentist during clinical examination [22].

### 4.5. From the Molecular Characteristics to the Clinical Practice

Saliva has immense potential as a critical diagnostic fluid for evaluating the overall microbiome, proteome, or genome sequences necessary for personalized monitoring [54]. Bioinformatics analyses and the insights they may reveal can lead the way to individualized evidence-based diagnostic and/or treatment options, including dietary modifications and education, enrichment of risk-assessment tools for deciding on the frequency of visits to dental practices [55], use of probiotic bacteria or prebiotics, or targeted antibiotics or other small molecules, and enrichment of risk-assessment tools for deciding on the frequency of visits to dental practices [54]. 

Bioinformatic tools can provide a straightforward and clinically meaningful interpretation. In this study, significant biological pathways involved in pathogenic processes, such as defense response to the bacterium, beta-defensin activity, proline-rich protein activity, and glycosylation, were deregulated in subjects with type 1 diabetes, highlighting the specific molecular characteristics integral to the cariogenic process of these individuals. Routine incorporation of microbiological and immunological parameters in daily clinical practice could enrich clinicians’ awareness of the underlying biological mechanisms and ecological considerations of a multifactorial disease such as dental caries. This would lead to a much-sought paradigm shift in oral care by guiding clinicians to focus on the etiological factors rather than the pathology, contributing to individualized and tailor-made (or patient-specific) preventive/treatment plans. Such developments will validate the usability of the gathered biological information for the patient’s benefit. Furthermore, they will propose modifications or further applications for actions that are practically relevant to their practice. The latter will enable individualized dentistry [54] and contribute to more successful primary prevention.

## 5. Conclusions

Poor metabolic control in adolescents with type 1 diabetes causes alterations in salivary protein composition and is significantly related to caries prevalence. Our results highlight saliva’s protective function by studying salivary proteome and confirming that salivary alterations impact caries processes. Furthermore, the downregulation of most differentially expressed proteins with a protective role against caries activity may explain the increased caries incidence in the studied population.

## Figures and Tables

**Table 1:** Clinical characteristics and caries index (DMFT) of the study population (* *p*-value < 0.05). Mean and standard deviation values (means ± SD) are reported.

Participants’ Characteristics	G1	G2	C
Age (yrs), mean (SD)	14.5 ± 1.7	14.1 ± 1.3	14.9 ± 1.8
Gender, *n* (Male/Female)	5/7	5/7	5/7
Time with DM1 (yrs)	5.8 ± 1.9	6.4 ± 2.8	-
HbA1c% (mmol/mol)	9.7 ± 0.7 * (83)	6.2 ± 0.4 * (44)	4.2 ± 0.4 * (22)
BMI (kg/m^2^)	22.9 ± 4.0	20.7 ± 5.0	24.3 ± 3.0
Blood Pressure (mmHg)	82 ± 5	79 ± 4	85 ± 5
Diastolic Blood Pressure (mmHg)	67 ± 3	63 ± 3	70 ± 4
Systolic Blood Pressure (mmHg)	113 ± 4	109 ± 3	114 ± 3
Total cholesterol (mg/dL)	165 ± 10	160 ± 12	168 ± 15
LDL cholesterol (mg/dL)	92 ± 6	88 ± 5	94 ± 8
Plaque Index (PI)	0.80 ± 0.05	0.60 ± 0.03	0.70 ± 0.02
Gingival Index (GI)	0.7 ± 0.2	0.5 ± 0.2	0.8 ± 0.1
DMFT	3.9 ± 0.7 *	0.9 ± 0.2 *	1.1 ± 0.3 *

**Table 2:** Selection of biologically relevant pathways, which could be associated with caries susceptibility. The proteins highlighted in red in each path are more biologically relevant to caries pathology.

**G1 vs. C L2R-p-DAVID**		
**Term**	**Proteins**	***p* value**
Secreted	**PRR4**, **AMY2B**, PRELP, **STATH**, ORM2, **BPIFA2**, C6ORF58, CHAD, **PRB2**, AMBP, **DCD**,** AMY1A**, **MUC19**, SPINK5, **DEFA3**, **IL36G**,** DEFA1**, **MUC5B**, BPIFB2, RBP4, ALB, HPSE, **DEFB4A**, LACRT, **S100A7**	1.79 × 10^−8^
GO:0042742~defense response to bacterium	IGLC7, **DCD, BPIFA2**, **DEFA3**, **MUC5B**, **DEFB4A**, **STATH**, LACRT	1.42 × 10^−6^
GO:0005796~Golgi lumen	**MUC19**, **DEFA3**, SDF4, PRELP, **DEFA1**, **MUC5B**, **DEFB4A**	1.98 × 10^−6^
GO:0019825~oxygen binding	ALB, CYP1A1, HBB, HBD, HBA1	2.00 × 10^−5^
signal peptide	**PRR4**, PSCA, **AMY2B**, PRELP, **STATH**, ORM2, **BPIFA2**, C6ORF58, CHAD, **PRB2**, SDF4, AMBP, DCD, **AMY1A**, **MUC19**, SPINK5, **DEFA3, DEFA1, MUC5B, **LRP1B, BPIFB2, RBP4, ALB, HPSE, PKHD1L1, **DEFB4A**, LACRT	3.07 × 10^−5^
IPR006080:Beta defensin-Neutrophil defensin	**DEFA3, DEFA1, DEFB4A**	2.49 × 10^−4^
Signal	**PRR4**, PSCA, **AMY2B**, PRELP, **STATH**, ORM2, **BPIFA2**, C6ORF58, CHAD, **PRB2**, SDF4, AMBP, **DCD**,** AMY1A**, **MUC19**, SPINK5, **DEFA3**, **DEFA1**,** MUC5B, LRP1B**,** BPIFB2**, RBP4,** ALB**, CYP1A1, HPSE, PKHD1L1, **DEFB4A**, LACRT	3.27 × 10^−4^
glycosylation site:N-linked (Glc) (glycation)	ALB, HBB, HBA1	3.54 × 10^−4^
Antimicrobial	**DCD**, **BPIFA2, DEFA3, DEFA1, DEFB4A**	3.84 × 10^−4^
Glycation	ALB, HBB, HBA1	4.04 × 10^−4^
hsa04970:Salivary secretion	**PRB2, MUC5B, STATH**, RYR3	0.001844339
calcium-binding region:2; high affinity	TCHH, S100P, **S100A7**	0.002649627
Calcium	PCLO, **AMY1A, AMY2B**, TCHH, SDF4, S100P, HPSE, RYR3, LRP1B, **S100A7**	0.003100924
Pyrrolidone carboxylic acid	**AMY1A, AMY2B, PRB2, ORM2**	0.004088861
GO:0050832~defense response to fungus	**DCD, DEFA3, DEFA1**	0.004847206
IPR000566:Lipocalin-cytosolic fatty-acid binding protein domain	RBP4, AMBP, ORM2	0.006685494
Defensin	**DEFA3, DEFA1, DEFB4A**	0.013921585
GO:0004556~alpha-amylase activity	**AMY1A, AMY2B**	0.017063442
IPR006046:Alpha amylase	**AMY1A, AMY2B**	0.0179226
GO:0050829~defense response to Gram-negative bacterium	**DEFB4A**, OPTN, **S100A7**	0.019204342
IPR016327:Alpha-defensin	**DEFA3, DEFA1**	0.021468961
IPR026086:Proline-rich protein	**PRR4, PRB2**	0.025002707
IPR002345:Lipocalin	RBP4, AMBP	0.028523881
Glycoprotein	PSCA, AMBP, WSCD1, **AMY1A**, SLC2A1, HBB, PRELP, HBA1, **MUC5B**, ORM2, LRP1B, FUT3, **BPIFB2**, C6ORF58, **BPIFA2**, CHAD, ALB, **PRB2, **CYP1A1, SDF4, HPSE, PKHD1L1, VN1R5, LACRT	0.029468179
GO:0050830~defense response to Gram-positive bacterium	**DEFA3, DEFA1, DEFB4A**	0.042886244
Phosphoprotein	SETD2, KMT2A, SYAP1, SLC2A1, HBB, HBD, CCNDBP1, **STATH**, GRAMD4, SELENBP1, PCLO, **PRB2**, XIRP1, SDF4, SPEG, DENR, ZC3H15, SZT2, CKAP2, FAM118A, **KRT7**, FRMD4A, ATP11B, UBE4B, **DEFA1**, HBA1, KRT74, LRP1B, BPIFB2, RRAGC, SUB1, ALB, ALPK3, FARSA, OPTN, TRIM56, CHMP5	0.044573527
**G2 vs. C L2R-p-DAVID**		
**Term**	**Proteins**	***p* value**
glycosylation site:N-linked (Glc) (glycation)	ALB, HBB, **APOA1**, HBA1, CFB	2.20 × 10^−7^
Glycation	ALB, HBB, **APOA1**, HBA1, CFB	7.69 × 10^−7^
Pyrrolidone carboxylic acid	ORM1, **AMY2B**,** PRB2, APOA2, **PRH1, KNG1	3.33 × 10^−4^
IPR026086:Proline-rich protein	**PRR4, PRB2, PRH1**	9.39 × 10^−4^
GO:0042742~defense response to bacterium	IGHG1, IGHG2, **DCD, MUC5B, DEFB4A**, LACRT	0.004284705
IPR002957:Keratin, type I	**KRT33B, KRT12, KRT9**	0.021047193
GO:0034190~apolipoprotein receptor binding	**APOA2, APOA1**	0.021526132
Antimicrobial	**DCD, DEFA1**, LYZ, **DEFB4A**	0.032341013
**G1 vs. G2 L2R-p-DAVID**		
**Term**	**Proteins**	***p* value**
Protease inhibitor	ITIH4, APP, **CST1**, SERPINA1, ITIH2, AMBP, SERPINC1, SPINK5, SERPING1, A2M, **CST5, CST4**	5.10 × 10^−11^
glycosylation site:O-linked (GalNAc...)	ITIH4, HPX, TF, ITIH2, AMBP, AHSG, SERPING1, PLG	9.29 × 10^−7^
Glycoprotein	APP, ITIH4, ORM1, SERPINA1, ITIH2, CFH, SERPINC1, PON1, LAMA3, SLC2A1, PLG, FURIN, A1BG, ORM2, PRH1, PKHD1, **C3**, IGHG3, C1QTNF3, IGHG4, HPX, **C6**, TTR, IGHG2, **C9**,** PRB2, **A2M, GC, CPA4, FGB, FGA, AMBP, AHSG, FGG, **APOA2, APOA1**, CP, TF, BPIFB1, SCGB2A1, ALB, CYP1A1, SERPING1, COL4A5, CFB	1.01 × 10^−5^
GO:0006956~complement activation	IGHG3, **C3**, IGHG4, **C6**, IGHG2, CFH, CFB	1.64 × 10^−5^
GO:0002020~protease binding	**CST1**, SERPINA1, SERPINC1, FURIN, A2M, **CST5, CST4**	2.37 × 10^−5^
Complement alternate pathway	**C3, CFH, C9, CFB**	2.99 × 10^−5^
GO:0006953~acute-phase response	ITIH4, ORM1, SERPINA1, AHSG, ORM2	9.67 × 10^−5^
GO:0045087~innate immune response	FGB, IGHG3, FGA, APP, IGHG4, C6, IGHG2, HMGB2, SERPING1, **IL36G**,** S100A7**	3.34 × 10^−4^
Complement pathway	**C3, C6, C9**, SERPING1	5.68 × 10^−4^
IPR026086:Proline-rich protein	**PRR4, PRB2, PRH1**	6.79 × 10^−4^
region of interest:Coil 1B	**KRT19, KRT12, KRT7, KRT75, KRT74**	6.84 × 10^−4^
GO:0044267~cellular protein metabolic process	FGA, APP, TTR, **APOA1**, FURIN, PLG	8.32 × 10^−4^
GO:0030674~protein binding, bridging	FGB, FGA, FGG, SPRR1A, OPTN	9.68 × 10^−4^
GO:1900026~positive regulation of substrate adhesion-dependent cell spreading	FGB, FGA, FGG, **APOA1**	0.001004558
glycosylation site:N-linked (Glc) (glycation)	ALB, **APOA1**, CFB	0.001008382
IPR001664:Intermediate filament protein	**KRT19, KRT12, KRT7, KRT75, KRT74**	0.001073524
Phosphoprotein	APP, SERPINA1, HMGB2, **SLC2A1**, PTPN23, UNC80, **PRB2**, DENR, DLAT, KCMF1, FGA, PCYT2, AHSG, CAD, DHPS, FGG, **APOA2**, CKAP2, **KRT7**, **APOA1**, ATP11B, TERF1, **KRT74**, CCDC80, ESRP2, SUB1, SPECC1L, ALPK3, OPTN, ITIH2, KMT2A, SERPINC1, FURIN, PLG, CCNDBP1, **STATH**, GRAMD4, PRH1, **CST4**, **C3**, RNF213, TTR, **C9**, TPR, XIRP1, HIVEP2, PCNT, **EPS15**, SASH1, PEX19, RAB27A, CP, MTOR, **KRT19**, TF, FUBP1, ALB, TACC2, TARDBP, FARSA	0.001509071
Glycation	ALB, **APOA1**, CFB	0.001739912
IPR027214:Cystatin	**CST1, CST5, CST4**	0.002464241
GO:0050829~defense response to Gram-negative bacterium	APP, HMGB2, OPTN, **S100A7**	0.004793789
IPR003054:Type II keratin	**KRT7, KRT75, KRT74**	0.011288975
IPR001500:Alpha-1-acid glycoprotein	ORM1, ORM2	0.011497863

**Table 3:** Selection of differentially expressed proteins involved in caries (fold change-up and down-regulation values obtained by iTRAQ). The proteins highlighted in red are more biologically relevant to caries pathology.

Proteins	G1. vs. C. Fold Change	G2. vs. C. Fold Change	G1. vs. G. Fold Change
**S100A7**	−1.688	−1.192	−1.416
**AMY2B**	−1.546	−1.382	−1.119
**DEFB4A**	−1.540	−1.300	−1.184
**DCD**	−1.434	−1.369	−1.048
**RBP4**	−1.418	1.003	−1.423
**BPIFB2**	−1.324	−1.174	−1.127
**PRB2**	−1.319	1.226	−1.617
**MUC19**	−1.272	−1.249	−1.018
**MUC5B**	−1.262	−1.184	−1.067
**IL36G**	−1.247	1.081	−1.349
**AMY1A**	−1.242	−1.058	−1.173
**STATH**	−1.235	−1.004	−1.229
CST3	−1.222	−1.130	−1.081
**APOA2**	−1.222	1.174	−1.435
CST5	−1.209	−1.008	−1.199
KRT9	−1.204	−1.165	−1.034
**BPIFB1**	−1.201	1.057	−1.269
APOA1	−1.198	1.247	−1.496
MUC7	−1.188	−1.236	1.040
SERPINC1	−1.166	1.058	−1.235
**CST4**	−1.162	1.046	−1.216
**CST1**	−1.149	1.053	−1.211
**C6**	−1.135	1.111	−1.261
**C3**	−1.120	1.074	−1.202
**C9**	−1.118	1.104	−1.235
**BPI**	−1.087	1.099	−1.196
**KRT77**	1.147	−1.025	1.175
**KRT19**	1.223	1.010	1.211
**DEFA3**	1.224	1.113	1.100
**DEFA1**	1.228	1.223	1.004
**SPRR1A**	1.255	1.049	1.197
**KRT74**	1.337	1.000	1.337
ATP11B	1.471	1.046	1.406
**PRR4**	1.497	−1.256	1.880
**ORM2**	−1.261	1.120	−1.412
**ALB**	−1.268	1.169	−1.482

## Data Availability

The data presented in this study are openly available and uploaded as Supplementary Files.

## Supplementary Materials

The following are available online: https://www.mdpi.com/article/10.3390/proteomes9030033/s1. Table S1: Peptides and transitions detected by MRM analysis; Table S2: all the identifications and statistically significant changes, criterion *p*-value of fold change, for all comparisons; Table S3: the proteins lists of the Venn Diagram; Table S4: all the pathways/biological processes across the three comparisons; Table S5: the list with the annotations in G1-C from STRING analysis; Table S6: Log2ratio and L2R_pvalues are presented for differentially expressed proteins involved in caries. Table S7: MRM versus iTRAQ Log2ratio values are presented.
