# Supplementary material for: Downregulation of Salivary Proteins, Protective against Dental Caries, in Type 1 Diabetes
Source: Proteomes. 2021 Jul 19;9(3):33. doi: 10.3390/proteomes9030033 (PMC8293329; doi:10.3390/proteomes9030033)
Supplement: Supplementary file 1 [file proteomes-09-00033-s001.zip › Supplementary Table 6.L2R values and L2R_pvalues.doc .pdf]

<u>Supplementary Table 5.</u> Selection of differentially expressed proteins involved in caries. Fold change, Log2ratio and L2R_pvalues are presented.

gene	G1.vs.C. L2R	G1.vs.C. L2R.pvalue	G1.vs.C. fold change	G2.vs.C. L2R	G2.vs.C. L2R.pvalue	G2.vs.C. fold change	G1.vs.G2 .L2R	G1.vs.G2. L2R.pvalue	G1.vs.G2 .fold change
S100A7	-0.755	5.33E-07	-1.688	-0.253	0.019	-1.192	-0.502	0.0001	-1.416
AMY2B	-0.629	3.03E-05	-1.546	-0.467	1.39E-05	-1.382	-0.162	0.225	-1.119
DEFB4A	-0.623	3.60E-05	-1.540	-0.379	0.0004	-1.300	-0.244	0.066	-1.184
DCD	-0.520	0.005	-1.434	-0.453	2.54E-05	-1.369	-0.067	0.617	-1.048
RBP4	-0.504	0.001	-1.418	0.005	0.960	1.003	-0.509	0.0001	-1.423
BPIFB2	-0.405	0.007	-1.324	-0.231	0.032	-1.174	-0.173	0.1928	-1.127
PRB2	-0.399	0.008	-1.319	0.294	0.006	1.226	-0.693	1.59E-07	-1.617
MUC19	-0.347	0.022	-1.272	-0.321	0.003	-1.249	-0.026	0.852	-1.018
MUC5B	-0.336	0.026	-1.262	-0.244	0.024	-1.184	-0.093	0.491	-1.067
IL36G	-0.319	0.035	-1.247	0.113	0.289	1.081	-0.432	0.001	-1.349
AMY1A	-0.313	0.039	-1.242	-0.082	0.448	-1.058	-0.230	0.082	-1.173
STATH	-0.304	0.045	-1.235	-0.006	0.964	-1.004	-0.298	0.024	-1.229
CST3	-0.289	0.056	-1.222	-0.176	0.102	-1.130	-0.113	0.398	-1.081
APOA2	-0.289	0.056	-1.222	0.232	0.029	1.174	-0.521	8.14E-05	-1.435
CST5	-0.274	0.071	-1.209	-0.011	0.921	-1.008	-0.262	0.048	-1.199
KRT9	-0.268	0.078	-1.204	-0.220	0.041	-1.165	-0.048	0.725	-1.034
BPIFB1	-0.264	0.082	-1.201	0.080	0.450	1.057	-0.344	0.009	-1.269
APOA1	-0.261	0.085	-1.198	0.319	0.002	1.247	-0.581	1.13E-05	-1.496
MUC7	-0.249	0.101	-1.188	-0.306	0.004	-1.236	0.057	0.656	1.040
SERPINC1	-0.222	0.143	-1.166	0.082	0.440	1.058	-0.304	0.021	-1.235
CST4	-0.216	0.154	-1.162	0.065	0.538	1.046	-0.282	0.033	-1.216
CST1	-0.201	0.187	-1.149	0.075	0.479	1.053	-0.276	0.037	-1.211
C6	-0.183	0.230	-1.135	0.152	0.153	1.111	-0.335	0.011	-1.261
C3	-0.163	0.285	-1.120	0.103	0.334	1.074	-0.266	0.045	-1.202
C9	-0.161	0.291	-1.118	0.143	0.179	1.104	-0.305	0.021	-1.235
BPI	-0.121	0.429	-1.087	0.136	0.201	1.099	-0.258	0.052	-1.196
KRT77	0.198	0.181	1.147	-0.035	0.749	-1.025	0.233	0.074	1.175
KRT19	0.291	0.050	1.223	0.015	0.883	1.010	0.276	0.035	1.211
DEFA3	0.292	0.049	1.224	0.155	0.146	1.113	0.137	0.292	1.100
DEFA1	0.296	0.046	1.228	0.290	0.006	1.223	0.006	0.950	1.004
SPRR1A	0.328	0.027	1.255	0.069	0.514	1.049	0.259	0.048	1.197
KRT74	0.419	0.005	1.337	0.000	1	1.000	0.419	0.001	1.337
ATP11B	0.557	0.001	1.471	0.065	0.541	1.046	0.492	0.0001	1.406
PRR4	0.582	9.79E-05	1.497	-0.329	0.002	-1.256	0.911	4.34E-12	1.880
ORM2	-0.334	0.027	-1.261	0.163	0.125	1.120	-0.498	0.0001	-1.412
ALB	-0.342	0.023	-1.268	0.225	0.034	1.169	-0.568	1.73E-05	-1.482
